# Where to Cross Over? Defining Crossover Sites in Plants

**DOI:** 10.3389/fgene.2018.00609

**Published:** 2018-12-12

**Authors:** Julia Dluzewska, Maja Szymanska, Piotr A. Ziolkowski

**Affiliations:** Department of Genome Biology, Institute of Molecular Biology and Biotechnology, Adam Mickiewicz University, Poznań, Poland

**Keywords:** meiotic crossover, recombination hot spot, double-strand break (DSB), heterozygosity, polymorphism (genetic), DNA methylation, plants, chromatin

## Abstract

It is believed that recombination in meiosis serves to reshuffle genetic material from both parents to increase genetic variation in the progeny. At the same time, the number of crossovers is usually kept at a very low level. As a consequence, many organisms need to make the best possible use from the one or two crossovers that occur per chromosome in meiosis. From this perspective, the decision of where to allocate rare crossover events becomes an important issue, especially in self-pollinating plant species, which experience limited variation due to inbreeding. However, the freedom in crossover allocation is significantly limited by other, genetic and non-genetic factors, including chromatin structure. Here we summarize recent progress in our understanding of those processes with a special emphasis on plant genomes. First, we focus on factors which influence the distribution of recombination initiation sites and discuss their effects at both, the single hotspot level and at the chromosome scale. We also briefly explain the aspects of hotspot evolution and their regulation. Next, we analyze how recombination initiation sites translate into the development of crossovers and their location. Moreover, we provide an overview of the sequence polymorphism impact on crossover formation and chromosomal distribution.

## Introduction

Crossover recombination lies in the center of sexual reproduction, providing physical connections between homologous chromosomes during meiosis. In most sexually reproducing eukaryotes these connections are required to ensure proper segregation of chromosomes during the first meiotic division (Moore and Orr-Weaver, 1997; Villeneuve and Hillers, 2001). To fulfill this requirement only one crossover per each chromosome pair is needed and many species regulate the crossover number to not exceed this lower functional limit (Mercier et al., 2015; Ritz et al., 2017). In fact, it was estimated that amongst nearly 50 eukaryotes belonging to different kingdoms, 80% of chromosome pairs have fewer than 3 crossovers (Fernandes et al., 2018b). Both indirect and direct data indicate that crossover rate is under selection in both directions (Ritz et al., 2017). The reasons for selection against crossover rate being too low are easy to understand: lack of crossover may lead to chromosome non-disjunction, which can yield in aneuploidy. The causes of constrains against too frequent recombination are less intuitive, as crossovers increase genetic diversity within population by breaking up haplotypes. However, recombination can also break association between beneficial alleles on the same haplotypes, which might lead to reduced progeny fitness (Otto and Lenormand, 2002). In fact, there is still not much empirical evidence that recombination is advantageous for natural population under selection (Otto, 2009). Benefits seem to emerge for finite populations in situations where selection varies over time and/or space (Otto, 2009). As these situations are not permanent, the crossover rate may evolve to be kept at low levels. Direct evidence that crossovers carry more *de novo* mutations than non-recombinant DNA molecules has been recently reported for human (Arbeithuber et al., 2015). In Arabidopsis, higher mutation rate was reported in regions proximal to crossovers (Yang et al., 2015). This would suggest that crossover repair is more mutagenic than other meiotic repair pathways. Moreover, recent results for Arabidopsis plants, where crossover rate was significantly increased by combining mutations of different anti-crossover factors (Fernandes et al., 2018b) and/or overexpression of pro-crossover factors (Serra et al., 2018b) indicated some fertility defects apparent even in the first generations. Detailed cytological investigation suggested that those defects are likely due to improper repair of a subset of recombination intermediates (Fernandes et al., 2018b). This would potentially result in dangerous accumulation of mutations in subsequent generations. It is possible, however, that the improperly repaired intermediates do not necessarily include or are not limited to crossovers, hence these findings cannot be considered as a prove of crossover genotoxicity.

Whatever are the reasons of restricted crossover numbers, this rises an interesting question: where to put the crossover to get the best from it. Historically, it was believed that crossover distribution is even – actually this assumption stands as a major basis of genetic (recombination) mapping (Sturtevant, 1913). But even in the very first work of Sturtevant (1913) it was suggested that the map distances are not just physical, but are some kind of joint function between length and “strength” over a region of chromosome. With time we have realized that the assumption on random crossover distribution is far from being accurate, though useful for many genetic approaches. We currently know that a large number of different factors is responsible for chromosomal distribution of crossovers. In this review, we discuss different levels of control for crossover chromosomal distribution with a special emphasis on DNA heterozygosity. Mammalian systems are very distinct in this respect, mainly due to the presence of mammalian-specific PRDM9 histone methyltransferase, which is a major determinant of crossover pattern in human and mice (Baudat et al., 2010; Parvanov et al., 2010). Therefore, we will specifically focus on factors determining crossover location in plants and support this view with extensive progress in understanding of the recombination-related processes, which has been achieved in budding yeast.

## General Information About Crossover in Plants

The initial step inducing meiotic recombination is the formation of programmed DNA double-strand breaks (DSBs) catalyzed by the conserved topoisomerase-like complex, SPO11/TPOVIBL (Keeney et al., 1997; de Massy, 2013; Robert et al., 2016; Vrielynck et al., 2016). Following formation, DSBs are resected to generate single-stranded DNA (ssDNA), which is bound by the RecA-related recombinases RAD51 and DMC1 (Mercier et al., 2015). As a result, nucleoprotein filaments are created and further invade a sister chromatid or a chromatid located on a homologous chromosome. This results in a displacement loop (D-loop), which could be further processed via second-end capture to form double Holliday junction (dHJ) between the two chromatids (Wang and Copenhaver, 2018). It has been accepted that DMC1, a meiosis-specific homologue of RecA protein, is responsible for interhomolog bias – an increased chance of repair using homolog chromatid (Schwacha and Kleckner, 1997; Kurzbauer et al., 2012). The resolution of duplexes formed between sister chromatid results in DNA molecules, which are undistinguishable from their parental molecules, as they do not differ in DNA sequence. Repair based on non-sister chromatids may proceed via either several various non-crossover or crossover pathways. In contrast to crossovers (COs), where large fragments of DNA are reciprocally exchanged between parental chromosomes, non-crossovers (NCOs) result in gene conversion without affecting the template. The decision, which of DSBs will mature into crossover, and which will be repaired by non-crossovers, is called crossover designation.

In most eukaryotes including plants, two types of crossover pathways exist. The major pathway, named ZMM after the budding yeast genes *ZIP1, ZIP2, ZIP3, ZIP4, MSH4, MSH5*, and *MER3*, results in 85–90% of crossovers (called class I crossovers) in Arabidopsis, maize and rice (Higgins et al., 2004; Mercier et al., 2005; Falque et al., 2009; Shen et al., 2012). This pathway is meiosis-specific and depends on synaptonemal complex (SC) formation (Lynn et al., 2007). It is believed that ZMM proteins act to stabilize the interhomolog recombination intermediates to promote resolution by crossover (Lynn et al., 2007). Class I crossovers show interference, i.e., occurrence of a crossover in one location on a chromosome reduces significantly a chance for a second crossover in adjacent region on the same chromosome. Interference is detectable over the scale of megabases in Arabidopsis (Lynn et al., 2007; Mercier et al., 2015). Although interference has a great impact on chromosomal distribution of crossovers, it will not be discussed in this review as there are numerous articles focusing specifically on this phenomenon (Wang et al., 2015; Sun et al., 2017).

The residual, non-ZMM crossovers (class II COs) are interference independent. The best-known player for class II COs is MUS81, an endonuclease which is able to process joint molecules (e.g., D-loops). The null mutation of *MUS81* reduces recombination by 10% in wild-type Arabidopsis plants and eliminates *ca.* 1/3 of the residual COs in *zmm* mutants (Berchowitz et al., 2007; Higgins et al., 2008a; Macaisne et al., 2011). This suggests that class II crossovers result from several different, partially redundant pathways. Opposite to ZMM pathway, the other pathways usually promote recombination intermediates resolution by non-crossovers.

## Impact of Dsb on Crossover Distribution

Distribution of DSBs could be considered as the first level of defining crossover sites. Obviously, crossover can occur only at a DSB site, hence blocking DSBs at one chromosomal location will automatically exclude this region from the pool of potential recombination sites. However, in most organisms including plants, the number of DSBs significantly exceeds the number of crossovers (Mercier et al., 2015). For example, there is about 150–300 DSBs in *Arabidopsis thaliana* producing only around 10 crossovers (Chelysheva et al., 2010; Kurzbauer et al., 2012; Choi et al., 2013). Similarly in maize, nearly 500 DSBs lead to the formation of about 20 crossovers (Anderson et al., 2003; Pawlowski et al., 2003; Sidhu et al., 2015). As a consequence, a crossover site is selected from a wide range of potential locations. Even though, CO distribution is significantly associated with high levels of DSBs in Arabidopsis, at least at a genome-wide scale (Choi et al., 2018). Similar correlation was not reported for maize, which could be due to a very different, heterochromatin-rich genome architecture of the former species (He et al., 2017).

### Recombination Initiation Hotspots in the Context of Chromatin Structure

In many eukaryotes DSBs occur in discrete, non-randomly distributed regions referred to as DSB hotspots. Their distribution at this scale is strictly dependent on chromatin structure, as meiotic chromosomes have specific architecture defined by chromatin loops protruding from proteinaceous axis (Blat et al., 2002; Borde and de Massy, 2013). Hotspots predominantly reside within loop regions, however, the machinery responsible for DSB formation, RMM complex (for budding yeast proteins REC114, MEI4, MER2), is physically located within the axis (Borde and de Massy, 2013). Interestingly, a strong anticorrelation between regions directly interacting with axis and with hotspot is observed, which suggests a repressing activity of some axis components. In addition, DSB hotspot regions frequently overlap with 5′ ends of yeast genes indicating that open chromatin states play a role in hotspot determining within the chromatin loops (Pan et al., 2011).

Trimethylation at histone 3 at lysine 4 (H3K4me3; to a lesser extent also dimethylation H3K4me2) has been found as an important determinant of DSB location in numerous eukaryotes and extensively investigated in budding yeast (Acquaviva et al., 2013; Sommermeyer et al., 2013). This histone mark, which is usually located at 5′-ends of genes, is also associated with high expression levels (Santos-Rosa et al., 2002). H3K4me3 is not considered as instructive for gene expression but rather as its consequence, which may also have a function in epigenetic memory (Howe et al., 2017). In contrary, H3K4 methylation seems to be very important for designation of DSB sites in budding yeast. First, it was observed that in the absence of H3K4me3 the number of meiotic DSB is significantly reduced (Borde et al., 2009). Borde et al. (2009) found also that elevated H3K4me3 levels near DSBs were independent of local transcription levels, replication and, more importantly, of DSB formation. Further work resulted in proposing a model, in which a PHD finger domain protein SPP1 recognizes H3K4me2/3 and tether the corresponding site of chromatin loop to chromosome axis by interaction with MER2 and other factors (Borde et al., 2009; Acquaviva et al., 2013; Sommermeyer et al., 2013). More recent report indicates that SPP1 protein adopts multiple roles in this process including meiosis-specific histone methyltransferase, dedicated for DSB designation (Adam et al., 2018). Whether similar mechanisms exist in plants remains unknown.

In plants, first analyses of DSB sites were performed indirectly by studying CO and NCO pattern within two recombination hotspots in *A. thaliana* (Drouaud et al., 2013). The authors noticed, however, that NCO tracts were relatively short and, due to limited polymorphisms, a large portion of the NCO events could not be detected. More recently, the DSB distribution was investigated at the genome-wide scale in maize using RAD51-ChIP (He et al., 2017) and in Arabidopsis using SPO11-oligos mapping (Choi et al., 2018). The first method uses antibodies to specifically precipitate DNA fragments associated with RAD51 – a protein that together with DMC1 binds to ssDNA formed at DSB site. SPO11-oligos mapping takes advantage from the fact that SPO11 protein covalently binds a short ssDNA fragment in the process of DSB formation; following SPO11 isolation from meiocytes, the DNA oligonucleotides may be extracted and sequenced to precisely identify DSB sites. Both analyses indicated relatively low correlation between DSBs and H3K4me3 at the chromosome and hotspot level, which is consistent with the data received from budding yeasts and mouse (Lange et al., 2016; Yamada et al., 2017a). However, correlation between COs and H3K4me3 was detected in both species (Choi et al., 2013; Shilo et al., 2015; He et al., 2017), suggesting that this chromatin mark may play similar role for loop tethering to chromosome axis as reported in yeast and mammals (Borde et al., 2009; Acquaviva et al., 2013; Sommermeyer et al., 2013; Adam et al., 2018). On the other hand, H3K4me3 is a universal chromatin mark associated with open chromatin structure, especially with transcription start sites, therefore its location next to CO sites may be purely coincidental.

Plant DSB hotspots have been estimated to be 1.2 kb (maize) and 0.8 kb (Arabidopsis) in size on average, and exhibit the most evident association with open chromatin structure defined as chromosome regions depleted in nucleosomes (He et al., 2017; Choi et al., 2018). This indicates that SPO11 acts in an opportunistic fashion, being able to cut different sequences as long as it has an access to DNA. This resembles the situation in budding yeast (Pan et al., 2011), but is very different from many mammals where DSB hotspots are mainly determined by PRDM9 meiosis-specific histone methyltransferase (Baudat et al., 2010; Lam and Keeney, 2015a). One clear negative correlation was observed between DSB hotspots and DNA methylation, visualized also in *met1* background DSB mapping, in which most of CG context DNA methylation is removed (Choi et al., 2018). In this case elevation of DSB levels clearly mirrored decrease in DNA methylation accompanied by reduction of nucleosome occupancy and slight increase in H3K4me3 in pericentromeric regions. At the fine scale, these changes in recombination initiation sites are specifically evident for some transposable element classes (Gypsy LTRs and EnSpm/CACTA), but not all (LINE and SINE), which clearly reflects alteration in CG methylation pattern in those elements (Choi et al., 2018). Furthermore, increase in DSBs was also observed in *kyp suvh5 suvh6* triple mutant, in which CHG and CHH methylation as well as H3K9me2 are strongly diminished (Underwood et al., 2018). Here the changes were mainly associated with elevation of DSB levels in centromeric repeats, such as *CEN180*. On the other hand, DNA methylation is usually associated with heterochromatin and closed chromatin structure (Zhang, 2008). Therefore, it is difficult to conclude whether the inhibition of DSB formation in methylated regions is a direct (e.g., due to physical obstacles during dHJ resolution) or indirect (e.g., secondary effects on chromatin structure) consequence of DNA methylation.

The number of DSBs tends to be modestly diminished in most repetitive sequences such as segmental duplications and repetitive transposons, however a subset of transposons (Stowaway elements in potato, and Helitron, Pogo/Tc1/Mariner DNA transposons in *A. thaliana*) are enriched in genomic regions spanning crossovers (Marand et al., 2017; Choi et al., 2018). The increased number of SPO11-oligos within Helitron, Pogo/Tc1/Mariner DNA repetitive transposons was observed when adjacent to immunity genes, which may contribute to enhanced favorable recombination within those regions (Choi et al., 2018).

When compared to plants, other eukaryotes have more pronounced DSB hotspots. In mammals this is due to the stringent PRDM9-dependent DSB patterning (Baudat et al., 2010; Parvanov et al., 2010). On the other hand, in budding yeast pronounced DSB peaks may be caused by extremely compact genome: from one side this contributes to significantly lower number of gene-related nucleosome-depleted regions (NDRs), which SPO11 tends to opportunistically bind to, and from the other side this results in a relatively high crossover pressure (de Massy, 2013). As it was mentioned, in plants, SPO11 hotspots are frequently found also in NDRs at the 3′-ends of genes, as well as in introns (Choi et al., 2018). This increases significantly the number of potential SPO11 targets in plant genomes, which may result in the more uniform recombination landscape.

### Transcription Factors (TFs)

In most eukaryotes including plants, DSB hotspots do not correlate with transcription in this mean that genes highly expressed in meiosis do not show higher DSB levels (Tischfield and Keeney, 2012; He et al., 2017; Choi et al., 2018; see however Yamada et al., 2017b). In some cases, however, DSB hotspot activity was connected with the binding of sequence-specific transcription factors. For instance, this has been reported at the *HIS4* locus in budding yeast and the *ade-M26* allele in fission yeast (White et al., 1991; Kon et al., 1997; Steiner et al., 2002). In fission yeast a heterodimeric basic-leucine-zipper transcription factor ATF1-PCR1 was found to recognize hotspot-specific DNA motifs *M26* leading to recruitment of DSB-machinery (Kon et al., 1997; Steiner et al., 2002). In the more recent genome-scale analysis of meiotic DSB landscape in *Schizosaccharomyces pombe*, the positive effect of hotspot activity was detected for less than a quarter of loci containing the motif showing that other factors contribute to the development of the recombination initiation hotspot (Fowler et al., 2014). In many of those loci DSBs seem to be symmetrically arrayed around the TF’s binding sites. Interestingly, about half of the identified *M26* hotspots showed increased DSB levels to one side of the motif while the other side exhibited strong transcription. The mechanism by which ATF1-PCR1 affects DSB formation remains elusive (Fowler et al., 2014). In summary, the study showed that binding of ATF1-PCR1 alone is not sufficient to target high levels of DSB formation nearby.

Although, the way how TFs affects recombination is ambiguous (Fowler et al., 2014; Zhu and Keeney, 2015), in some cases (*HIS4* and *PHO5* in budding yeast) the effect of TF is achieved by changing DNA accessibility in the hotspot region (Wu and Lichten, 1995). Therefore, it has been proposed that transcription factor-induced chromatin modifications underlie activation of recombination breakpoints (Hirota et al., 2008; Wahls and Davidson, 2010). The activating chromatin modifications are likely to belong to different types, however, formation of nucleosome-depleted regions along with histone acetylation and methylation at specific positions are considered as the most universal (Hirota et al., 2008; Getun et al., 2017).

An interesting observation is the formation of ATF-PCR1 heterodimer in response to osmotic stress, which in turn triggers meiosis in fission yeast. In budding yeast activation of *HIS4* DSB hotspot requires binding of RAP1, BAS1 and BAS2 transcription factors (White et al., 1993), and a more recent studies showed that a number of other DSB hotspots is dependent on these factors (Mieczkowski et al., 2006; Zhu and Keeney, 2015). Expression of those TFs is strictly linked with cell starvation that also induces meiosis. Thus, the transcription factors involved in preparation for stress response in fission and budding yeasts seem to be reutilized for DSB hotspot generation, resulting in an increased recombination rate. This coincidence could be beneficial from evolutionary point of view. Whether similar mechanisms exist in other organisms remains unknown and there are no clear examples that DSB hotspots might be regulated by TFs in higher eukaryotes. However, existence of similar TF-related modifiers, which would conditionally activate specific recombination hotspots, is an exciting possibility, especially in plants which show gene-located recombination hotspots. For instance, one could easily imagine potential benefits of targeting meiotic recombination nearby genes responsible for resistance to pathogen in biotic stress conditions. Indeed, some R-genes exhibit elevated recombination frequency, which was exemplified both at the historical (based on coalescent analysis) (Choi et al., 2013) and experimental data (Choi et al., 2016; Serra et al., 2018a).

Interestingly, the most important factor that determines recombination hotspot location in many mammals, PRDM9, emerged from a metazoan-specific family of TFs (Vervoort et al., 2016). In this case, however, the PRDM transcription factors are involved in a wide variety of functions during animal development but not in stress response, suggesting an accidental capturing of a new biological function by the TF. PRDM9 possesses an array of zinc-fingers, by which it recognizes specific DNA motifs and incorporates classical H3K4me3 and H4K36me3 marks defining recombination hotpots (Baker et al., 2015). It is currently unknown why modifications by PRDM9 have stronger effect on defining DSB sites than promoter-located H3K4me3 marks, which are formed by other methyltransferase complexes (Brick et al., 2012), however, recent data indicate that concentration of marks may play a role (Diagouraga et al., 2018).

### Local Base Composition and DNA Motifs

Local base composition, especially GC-rich regions, was found to be an important factor controlling distribution of recombination initiation hotspots in many organisms, from yeasts to mammals and plants (Blat and Kleckner, 1999; Gerton et al., 2000; Myers et al., 2008; Smeds et al., 2016; He et al., 2017). Several explanations for this phenomenon have been proposed including a higher susceptibility for recombination machinery in GC-enriched regions (Petes, 2001) and biased repair of mismatches in recombining regions toward G/C pairs (Birdsell, 2002). Recent work in budding yeast shows that incorporating GC-rich sequence into *URA3* hotspot significantly elevates meiotic and mitotic recombination rate (Kiktev et al., 2018). This would suggest that at least in this case the first hypothesis is correct. This relationship may be a consequence of DSB-formation dependence on chromatin structure, especially nucleosome positioning and specific histone modifications including H3K4me3, which is important for tethering of chromatin loops to chromosome axis.

High GC content has been found also in maize DSB hotspots (He et al., 2017). However, similar relationship was not reported for Arabidopsis (Choi et al., 2018; Underwood et al., 2018). This is surprising in the context of similarities between yeast and plant recombination hotspots, as in both cases nucleosome-depleted regions and open chromatin structures are recalled as the most characteristic features (Choi and Henderson, 2015). However, one should consider significant differences between the structure of Arabidopsis and maize genome: while the first one is very small and compact (0.12 Gb), deprived of transposable elements (TEs) and gene-rich (one gene per 4.5 kb, on average) (The Arabidopsis Genome Initiative, 2000), the other is extremely large (2.3 Gb), TE-rich, with a gene-island organization (single genes separated by very long stretches of non-coding repetitive sequences) (Rodgers-Melnick et al., 2016). As many TE sequences are relatively AT-rich, which is usually considered as a way to escape from silencing via RNA-dependent DNA methylation, from evolutionary perspective it might be beneficial to allocate hotspot in GC-rich regions, which more often belongs to genes. From this point of view, it would be interesting to check the methylation pattern of maize recombination initiation hotspots.

A closer insight into maize recombination landscape indicates that in fact the high GC content of DSB hotspots corresponds to a 20-bp-long sequence motif (He et al., 2017). The motif is present in more than 70% of genic hotspots, however, cannot be detected in repetitive DNA hotspots. In the same work the authors also described a crossover-associated motif, which is similar to DSB motif, however, contains overrepresentation of ‘C’ over ‘G’ (He et al., 2017). Similar, C-rich motifs have been described in Arabidopsis as crossover-associated (Choi et al., 2013; Wijnker et al., 2013; Shilo et al., 2015), although DSB-associated motifs seem to be rather AT-rich (Choi et al., 2018). All those findings suggest that it is not the GC content *per se* that is responsible for higher DSB formation or crossover formation, but instead the effect is related to specific sequence motifs which are likely recognized by recombination machinery. In addition, as AT-rich regions are known to exclude nucleosomes, it is suggested that those sequences are associated with more open chromatin structure affecting SPO11 accessibility and resulting in elevated DSB levels (Choi et al., 2018).

### Effects of Regulatory Circuits on DSB Distribution

**FIGURE 1:**
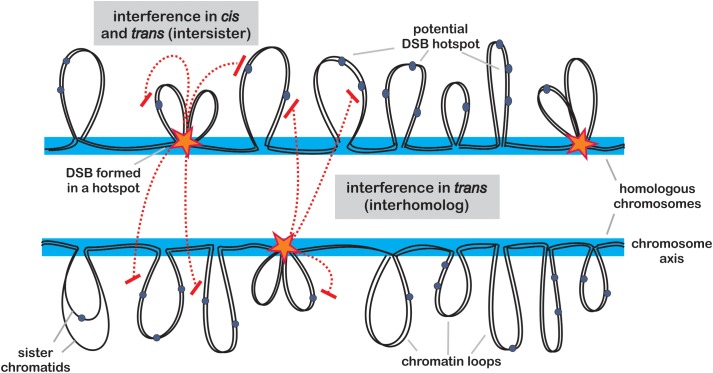
Model of DSB formation and control. Potential hotspot sites are located within chromatin loops (gray) protruding from chromosome axis (blue). Activation of a hotspot (star) requires tethering of the loop to the axis, where SPO11-containing protein complexes are deposited. Once a hotspot is activated, it communicates to other potential hotspots located on the same chromatid (in *cis*) or on its sister chromatid (in *trans*) causing their inhibition (red dashed lines). This process of positive DSB interference is dependent on ATM (TEL1) and ATR (MEC1) kinase signaling pathways and acts on distances of 30–100 kb in budding yeast. In addition, another form of interference inhibits formation of DSBs in potential hotspots located in the corresponding regions on homologous chromosome.

Beside chromatin landscape, which can be considered as relatively stable determinant of DSB pattern, a number of regulatory mechanisms actively affect the distribution of DSB hotspots in a cell-to-cell manner. DNA damage-response kinases TEL1^ATM^ and MEC1^ATR^ play a crucial role in this regulation. These proteins are also responsible for DSB interference (Xu and Kleckner, 1995; Lange et al., 2011; Lukaszewicz et al., 2018). Similar to crossover interference, DSB interference reduces the likelihood of DSB formation next to already formed DSB on the chromosome (Garcia et al., 2015). This phenomenon has been examined in details in budding yeast, and it appears to act only at short distances, usually below 100 kb, and is controlled mainly by TEL1^ATM^ (Robine et al., 2007) (Figure 1). It is believed that DSB interference is important to prevent formation of multiple DSBs in the same chromatin loop, which would be hazardous for genome stability (i.e., may cause chromosomal rearrangements). An interesting consequence of DSB interference is hotspot competition. Generation of an artificial DSB hotspot results in reduction of activity of surrounding hotspots, and the opposite can be observed when a hotspot is removed (Wu and Lichten, 1995; Pineda-Krch and Redfield, 2005; Robine et al., 2007; Acquaviva et al., 2013; Cooper et al., 2016).

In addition, another form of DSB interference has been detected in yeast, acting in *trans* - between sister chromatid or between homologous chromosomes (Xu and Kleckner, 1995; Rocco and Nicolas, 1996; Zhang et al., 2012) (Figure 1). This mechanism is controlled by TEL1^ATM^ and its partner MEC1^ATR^, and may act to reduce a chance of two DSBs occurring parallelly in corresponding chromosomal regions on both chromosomes, which would result in difficult to repair, complex double recombination event (Cooper et al., 2016). Recent work shows that MEC1^ATR^ is less sensitive toward DSB numbers than TEL1^ATM^, therefore it gets activated later during prophase I progression (Joshi et al., 2015). Early DSBs lack homolog bias, however, with progression of meiosis, subsequent MEC1^ATR^-dependent DSBs are more likely to result in interhomolog COs (Joshi et al., 2015). Currently, it is not clear whether the same mechanisms exist in plants, however, as the DNA damage-response kinases are conserved among all eukaryotes, it seems very probable. In Arabidopsis, null mutants of the TEL1^ATM^ and MEC1^ATR^ homologs, ATM (ataxia telangiectasia mutated) and ATR (ATM and RAD3-related), show significantly decreased fertility, and their double mutant exhibit extensive chromosome fragmentation in meiosis, which results in complete sterility (Garcia et al., 2003; Culligan and Britt, 2008). This indicates that those proteins play an important role in meiotic DSB repair, beside their functions in somatic DSB repair (Garcia et al., 2003; Culligan and Britt, 2008).

Besides ATM/ATR signaling, there are also additional pathways, which are involved in DSB formation. For example, continued DSB formation on unsynapsed chromosomes was observed in male mice with a lowered SPO11 dosage (Kauppi et al., 2013). This indicates that homolog engagement is acting to shut off DSB formation. There are reports suggesting that a similar mechanism can exist also in nematodes and involves meiotic cohesin component REC8 (Hayashi et al., 2007). In the more detailed study on the yeast model, it has been found that the phenomenon is ZMM-dependent with ZIP3 being involved in inhibition of DSBs after homolog engagement (Thacker et al., 2014). Therefore, though at the moment it is not clear whether the same mechanism acts in different species, it is very likely that homolog engagement is an additional level of DSB control conserved in eukaryotes.

### Evolution of Recombination Initiation Site Pattern

For a long time, theoretical studies on DSB hotspot evolution enforced a view predicting fast erosion of hotspot sequence leading to its rapid extinction (Boulton et al., 1997; Calabrese, 2007; Coop and Myers, 2007; Latrille et al., 2017). This “hotspot paradox” hypothesis was based on the assumption that a biased gene conversion occurs at a hotspot site, in which the broken chromosome copies DNA sequence from its uncut homolog. In consequence, the recombinationally active allele is replaced with its less active homolog, which results in its overrepresentation in progeny. Even in species where conversion tracts are very short and cannot significantly affect allele frequency (e.g., in Arabidopsis, Drouaud et al., 2013; Wijnker et al., 2013; Li et al., 2015), the active hotspot allele may be rapidly removed from the population simply by accumulation of point mutations. This is because recombination machinery seems to have a mutational effect, at least when crossover recombination is investigated (Lercher and Hurst, 2002; Arbeithuber et al., 2015; Yang et al., 2015). Mutations that reduce or eliminate hotspot activity will be consequently fixed in the population, whereas mutations activating hotspots will be removed.

**FIGURE 2:**
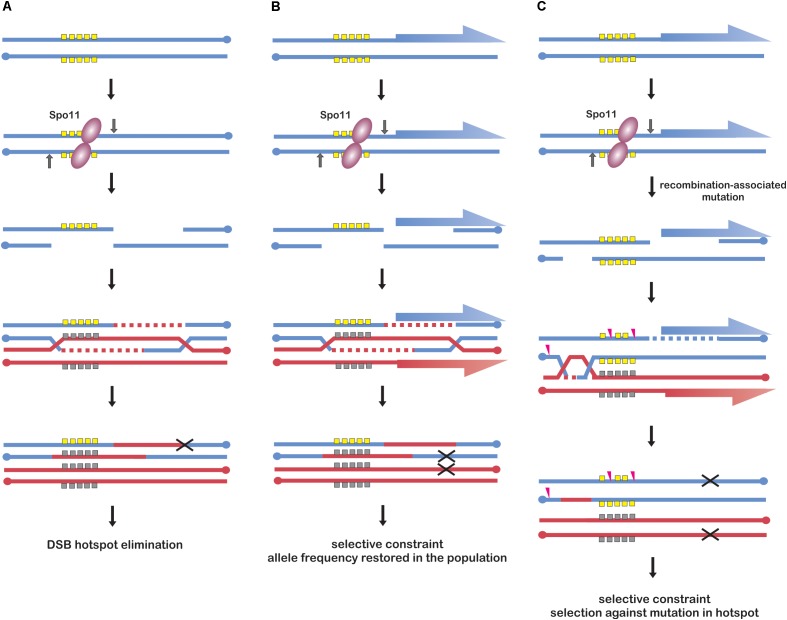
Models of recombination initiation hotspot evolution. **(A)** Frequent DSB formation in a strong hotspot allele (yellow squares) leads to increased conversion and rapid elimination of the allele from the population (black “X” symbol). This scenario is possible when the hotspot has no additional functions under selection, e.g., in human genome. Models **(B,C)** illustrate promoter-located hotspots; genes are indicated as blue and red half-arrows. **(B)** Frequent DSB formation in a hotspot allele (yellow squares) leads to its conversion to another allele (gray squares), however, selective constraint from its effect on promoter function enables its maintenance in the population. The selective constraint causes that particular hotspot alleles maintain comparable DSB activity. This model is likely to occur in yeast. **(C)** Frequent DSB events in a hotspot allele (yellow) result in an increased mutation rate leading to its erosion (pink arrowheads), however, selective constraint from its effect on regulatory elements enables maintenance of different original alleles in the population. Models **(B,C)** are not mutually exclusive and are likely to coexist in Arabidopsis.

Meiotic drive from biased conversion was reported in human (Jeffreys and Neumann, 2002), in which SPO11 is targeted to hotspots by PRDM9 protein. PRDM9 targets SPO11 to sites without additional biological functions, which are therefore released from evolutionary constrains other than those, which are recombination-related. Hence, PRDM9 recognition motifs can be subjected to meiotic drive from biased gene conversion leading to their rapid elimination from the population (Baker et al., 2015) (Figure 2A). As PRDM9-recognition motifs constantly disappear from the population, a new version of PRDM9 needs to evolve new combination of zinc-fingers to recognize novel motifs. For this reason PRDM9 belongs to the fastest evolving genes (Ponting, 2011). This phenomenon is observed in many species containing PRDM9-determined recombination initiation hotspots (Myers et al., 2010; Tiemann-Boege et al., 2017).

However, plants lack PRDM9 protein or analogous system. Plants hotspot arrangement resembles more that of yeast, with recombination initiation hotspots located mostly in nucleosome-depleted regions of promoters (Figure 2). Recent work in budding yeast investigated DSB hotspot location between several different strains and the comparison indicated that yeast hotspots are surprisingly stable (Lam and Keeney, 2015b). Moreover, when hotspot heat was compared, yeast hotspots from different strains appeared to be more similar than human hotspots between men sharing the same PRDM9 alleles, despite much greater sequence diversity (Pratto et al., 2014; Lam and Keeney, 2015b). Similarly, in vertebrate species lacking PRDM9 protein, e.g., birds, crossover hotspots seem to be highly conserved and it is likely that the same is true for recombination initiation hotspots (Singhal et al., 2015). The possible explanation of high hotspot conservancy in yeast could be the selective constraint for additional functions of the underlying sequences, which are usually related to their promoter locations (Nicolas et al., 1989; Pan et al., 2011; Lam and Keeney, 2015b) (Figure 2B). Analogous explanation has been proposed for evolution of avian recombination hotspots (Singhal et al., 2015). Answering the question whether similar, non-paradoxical stability of DSB landscape exists also in plants would require further work, which is now possible with newly developed DSB-mapping approaches in plant systems (He et al., 2017; Choi et al., 2018). However, frequent location of recombination initiation hotspot in gene promoters in plants suggests analogous situations, including also elimination of mutated alleles (Figures 2B,C). In this context, it would be interesting to compare promoter-located hotspots with those located in intergenic regions or transposable elements: according to the proposed scenario, they should evolve much faster/should have shorter life time than promoter-located hotspots.

### Recombination Initiation Sites at the Chromosome Scale

Although techniques enabling precise, sequence-based mapping of recombination initiation sites have been developed only recently, identification of those events on the chromosome scale has been possible for a long time based on observation of recombination nodules (RN) (reviewed in Anderson and Stack, 2005). Plant studies of those structures resulted in identification of two types of RN: early (ENs), which are associated with SC from leptotene into pachytene, and late (LNs), observed from pachytene into diplotene. The number of RNs declines in the course of meiosis, therefore ENs are thought to correspond to some stages of DSB repair, while LNs should represent those recombination events that are being repaired via crossover pathways (Zickler and Kleckner, 1998). ENs are randomly distributed along chromosomes at zygotene and do not show interference except at very short distances (in maize ≤ 0.2 μm) (Stack and Anderson, 2002). Nevertheless, ENs tend to be more frequent in distal euchromatic regions of bivalents than in proximal, heterochromatic segments, and the highest concentration of ENs occurs at synaptic forks suggesting their role in homologous synapsis (Stack and Anderson, 2002).

More specific features of recombination initiation sites became apparent with the emergence of high-resolution techniques. At the chromosome scale DSBs exhibit surprisingly uniform pattern of distribution, which has strong negative correlation with nucleosome occupancy in Arabidopsis (Spearman ρ = -0.7 or -0.95 for chromosome arms and pericentromeres, respectively) (Choi et al., 2018). The only regions that show dramatic suppression of recombination initiation are pericentromeres, which also reflects the effect of nucleosome occupancy, as in Arabidopsis heterochromatin is limited almost exclusively to those parts of the genome (The Arabidopsis Genome Initiative, 2000; Simon et al., 2015). All other tested correlations appear to be secondary or derivatives of the nucleosome positioning, especially when compared between chromosome arms and pericentromeres. For example, H3K4me3 was positively correlated with SPO11-oligos in the pericentromeric regions, but negatively in chromosome arms (Spearman ρ = 0.85 or – 0.82, respectively) (Choi et al., 2018). Similar observations were made in maize as assessed by RAD51 ChIP-seq with this difference that DSBs were abundant also in pericentromeric regions (He et al., 2017). Maize genome, however, is characterized by many more TE-rich heterochromatic regions, which show high DNA methylation levels and spread more uniformly along the chromosomes, with the majority of genes (∼85%) positioned within 1 kb of transposons (Schnable et al., 2009; Rodgers-Melnick et al., 2016). Other reasons of differences between Arabidopsis and maize patterns of recombination initiation sites may originate from technical issues applied.

### CO Homeostasis

An interesting phenomenon, which links recombination initiation sites (DSBs) with crossover control, is CO homeostasis. This regulatory pathway, which was observed in budding yeast, *Caenorhabditis elegans* and mice, causes no change in CO numbers even when substantial variation in DSB number is induced (Martini et al., 2006; Rosu et al., 2011; Cole et al., 2012; Yokoo et al., 2012). Recent experiments indicated a limit in CO homeostasis in maize: CO control was robust as long as one crossover per chromosome pair was ensured, but above this threshold the number of COs was linearly correlated to the number of DSBs (Sidhu et al., 2015). In Arabidopsis *fas1* mutant, in which a significant increase in DSB number is observed, no change in COs was reported (Varas et al., 2015). However, in hypomorphic *A. thaliana* *spo11* mutants, the reduction in DSB number resulted in proportional, though smaller, reduction in COs (Xue et al., 2018). Those two results are not necessarily mutually excluding, as it is possible that, similarly to maize, in Arabidopsis and other plants CO homeostasis works robustly only in some ranges. Interestingly, a dramatic change in CO distribution was observed in the hypomorphic *spo11* mutants, with substantial reduction of recombination in pericentromeres (Xue et al., 2018).

## Factors Influencing Spatial Distribution of Crossover

Meiotic DSBs may be repaired by several different mechanisms (see Introduction) and only a minority of them become crossovers (Mercier et al., 2015). The decision, whether the break should be repaired as crossover or non-crossover, is made based on a number of factors, which are largely unknown. Some factors influencing this decision, like modification by stress, have been extensively reviewed in recent works (Modliszewski and Copenhaver, 2017; Morgan et al., 2017) and therefore this topic will not be discussed. However, it should be emphasized that significant differences are observed between recombination initiation site distribution (DSB distribution) and crossover distribution.

In plants, NCO repair leads to minimal exchanges of genetic information between parental genomes, because the conversion tracts are very short and often undetectable (Lu et al., 2012; Sun et al., 2012; Drouaud et al., 2013; Wijnker et al., 2013). Hence, CO remains a major cause of genetic material reshuffling, important for variation in natural plant populations.

### Chromosome Level

Similarly to other eukaryotes, the crossover formation in plants is strongly biased toward euchromatic regions, in the contrast to CO inhibition at heterochromatin (Copenhaver et al., 1999; Giraut et al., 2011; Salomé et al., 2011; Mayer et al., 2012; Tomato Genome Consortium, 2012; Yelina et al., 2012; Rodgers-Melnick et al., 2016; He et al., 2017). Crossover suppression in the proximity of centromeres is important for fertility, as recombination events at those sites have been associated with chromosome segregation errors and aneuploidy (Rockmill et al., 2006; Stewart et al., 2008). Plant genomes show strong correlation between gene density and the distribution of genetic crossovers (Erayman et al., 2004; Choi et al., 2013; Wijnker et al., 2013; Rodgers-Melnick et al., 2015). Such fact is especially true for large genome species, like cereals, where crossover events are dramatically skewed toward the distal euchromatic, gene-rich regions of chromosomes. For instance, analyses in barley (Künzel et al., 2000; Mayer et al., 2012; Phillips et al., 2013), maize (Anderson et al., 2003; He et al., 2017) and wheat (Saintenac et al., 2009; Darrier et al., 2017) show elevations in CO number and gene density in subtelomeres and, at the same time, there is a decrease in recombination events and gene number in centromeric and pericentromeric regions (Schnable et al., 2009; Mayer et al., 2012; Tomato Genome Consortium, 2012; Higgins et al., 2014). Cytological studies in barley revealed that observed skewed chiasma distribution reflects polarization in the spatiotemporal initiation of recombination, chromosome pairing, and synapsis. Meiotic progression in distal chromosomal arms occurs in coordination with the chromatin cycles, whilst in interstitial and proximal regions meiotic initiation occurs later, is not coordinated with the cycles, and rarely progresses to form chiasmata. This early meiotic initiation is linked with euchromatic DNA, whilst late replication is observed at heterochromatin, in the centromeric and pericentromeric regions (Higgins et al., 2012).

Association of crossovers with a genic part of the plant genome may be a consequence of a specific, open chromatin structure within promoters and at the 3′-ends of genes, which authorizes the access to DNA. This could be especially important during meiosis, where chromatin is largely condensed and therefore inaccessible for recombination machinery. In concordance with this hypothesis is the intragenic pattern of COs: meta-analyses of both Arabidopsis and maize crossovers showed their underrepresentation within gene bodies and elevation at core promoters and at 3′-UTRs (Wijnker et al., 2013; Rodgers-Melnick et al., 2016). This points out the opportunistic feature of recombination complexes in plants, which is likely a consequence of SPO11 preferences.

Interestingly, the pattern of crossover distribution in some details is different from DSB distribution. For instance, in maize, large DSB numbers are formed in heterochromatic regions, however, they have very low chance for being repaired by crossover (He et al., 2017). This indicates that particular hotpots may significantly differ in CO/NCO ratios. In Arabidopsis, DSBs were not detected within pericentromeric regions, however, their distribution, though correlated to crossovers, shows also some significant differences: DSB levels exhibit more even distribution along the chromosomes than crossovers (Choi et al., 2018). It is unclear what is the reason of this discrepancy, but one factor could be a different genetic material used to achieve both datasets: SPO11-oligos used to map DSBs were obtained from the complemented *spo11-1* mutant line in homozygous Col-0 background, while crossover maps were achieved based on recombination events in Col x Ler F_1_ plants, two different *A. thaliana* accessions. In consequence, the crossover landscape is shaped by multiple modifiers of recombination, especially the impact of heterozygosity pattern (see below). Investigation of DSBs by SPO11-oligos mapping in the Col x Ler or Ler alone genetic background would provide us a deeper understanding of the relationship between recombination initiation sites and crossover formation. Another explanation could be spatiotemporal characteristics of DSB formation and crossover repair. DSBs are formed within relatively long time during prophase of meiosis I, in parallel to chromosome condensation, however, it is thought that only the late events result in crossover repair (Anderson and Stack, 2005; Joshi et al., 2015). In consequence, the chromatin stage of early and late DSBs may be different, which would result in observed dissimilarities. This hypothesis requires more detailed analysis of chromosome condensation.

### Heterochiasmy

Sex differences in recombination, known as heterochiasmy, are a widespread phenomenon described for the first time more than a century ago (Lenormand and Dutheil, 2005). Since that time an extensive set of data from many different species was collected indicating that heterochiasmy is a common feature of eukaryotes, including plants (Lenormand and Dutheil, 2005). Several hypotheses were proposed, however, none of them satisfactorily explain the variation in heterochiasmy in all species (Hedrick, 2007). This may indicate that the causes of heterochiasmy may be different in different taxa.

Heterochiasmy refers to both differences in recombination frequency and its spatial distribution along the chromosomes. We are especially interested in the second aspect of differences in recombination between sexes. In plants, this was extensively studied in Arabidopsis, where dramatic differences in COs, with a very significant, 1.8x higher rates in male compared to female meiosis were observed (Vizir and Korol, 1990). Very similar data were collected by Giraut et al. (2011) who investigated in details chromosomal distribution of crossovers. Although male crossovers are slightly elevated along the whole genome in almost all intervals tested, statistically significant differences were reported only for intervals in subtelomeric regions, and they encompass for the majority of differences in CO rates between sexes (Giraut et al., 2011). Interestingly, the ratio of the male vs. female genetic map length is very similar to the ratio of total SC length between male and female meiosis (Drouaud et al., 2007; Giraut et al., 2011). Similar observations were also made in other species, including mice, *Drosophila*, human and zebrafish (Kleckner et al., 2003). In *C. elegans* modification of SC length by a mutation in subunits of condensin results in increased CO rates (Mets and Meyer, 2009). Therefore, it is tempting to speculate that the length of SC determines the crossover number. This hypothesis is supported by recent findings in mice, where a map of recombination initiation sites (based on DMC1 binding) for males and females were achieved (Brick et al., 2018). The authors provided evidence that DSB frequency is not the driver of sex differences in distal crossovers in this species.

Recently, analysis of sex patterns in COs was carried out in maize (Kianian et al., 2018). The authors did not report significant differences in the spatial distribution of COs at the global scale, however, male and female COs differ at the fine scale, in their locations relative to transcription start sites in gene promoters. Differences were also observed with the respect to chromatin marks, including nucleosome occupancy and H3K4me3 (Kianian et al., 2018). This indicates that the sex specific features of crossover distribution could be observed even in species, where the global CO landscape remains the same between male and female meiosis. The mechanisms responsible for those differences are rather complex and currently poorly known. It would be interesting to study how sex differences in COs affect population structure and genome evolution.

### DNA Methylation

In plants, DNA methylation occurs in CG, CHG and CHH sequence context (where H = A, T or C) (Law and Jacobsen, 2010). CG methylation is maintained during replication by Methyltransferase1 (Met1) with the help of SWI/SNF chromatin remodeling protein Decreased DNA Methylation1 (DDM1) (Vongs et al., 1993; Saze et al., 2003; Stroud et al., 2012; Zemach et al., 2013). Non-CG methylation is maintained by Chromomethylase2 (CMT2), Chromomethylase3 (CMT3) and Domains Rearranged Mathylase2 (DRM2) (Lindroth et al., 2001; Malagnac et al., 2002; Cao et al., 2003; Du et al., 2012; Stroud et al., 2012). The methylation in non-CG contexts require methylation of histone H3K9 by SET domain methyltransferases (Jackson et al., 2002; Malagnac et al., 2002; Johnson et al., 2007; Du et al., 2014). As the two processes are linked, the non-CG methylation mutants exhibit also reduced H3K9me2.

In Arabidopsis and maize, genome-wide analyses of CO hotspots show low levels of DNA methylation (Choi et al., 2013; Rodgers-Melnick et al., 2015; Kianian et al., 2018). Studies of non-CG methylation mutants, *met1* and *ddm1*, documented that epigenetic crossover remodeling decreases within pericentromeric region and simultaneously increases in gene-rich chromosome arms in *Arabidopsis thaliana* (Mirouze et al., 2012; Yelina et al., 2012; Melamed-Bessudo and Levy, 2012). This is somehow surprising as significant loss of CG methylation in pericentromeres should result in elevation of recombination in those regions. The mutants did not significantly alter the total number of COs, but rather led to their redistribution along the chromosomes, which suggests the involvement of CO interference (Colomé-Tatché et al., 2012; Melamed-Bessudo and Levy, 2012; Mirouze et al., 2012; Yelina et al., 2012, 2015). In a more recent report Yelina et al. (2015) compared the effect of two crossover pathways in the *met1* mutant background and concluded that crossover remodeling is due to the interfering pathway. They proposed that loss of DNA methylation either changes relative timing of DSB formation between arms and pericentromeres, or reduces the chance of crossover designation in the proximity of a centromere. As a consequence of crossover interference, the chromosome arms receive additional COs compared to wild type (Yelina et al., 2015).

Interestingly, very different effect was observed in mutants causing loss of CHG and CHH methylation. Underwood et al. (2018) observed increased CO rate in pericentromeres with simultaneous moderate reduction in chromosome arms. SPO11-oligos mapping revealed a significant increase in DSB levels within centromeres but not adjacent pericentromeric regions in the H3K9me2/non-CG pathway mutant showing that the effect on crossover is not a simple consequence of the DSB level change. Choi et al. (2018) corroborated that a similar increase in DSBs can be observed also in the *met1* mutant. Thus, the two types of DNA methylation, CG and non-CG, are able to trigger similar change in DSB pattern, but have almost opposite consequences on CO distribution. The authors proposed that while both CG and non-CG methylation inhibit DSB formation, only non-CG methylation and/or H3K9me2 inhibit crossover (Underwood et al., 2018). In concordance with this hypothesis, euchromatic crossover hotspots in Arabidopsis can be silenced via RNA-directed DNA methylation pathway, which causes both CG and non-CG methylation as well as the increase in H3K9me2 mark (Yelina et al., 2015). It would be interesting to elucidate which of this epigenetic modification is so important for crossover formation.

## Effects of Heterozygosity on Crossover Distribution

Mismatches between DNA sequences in homologous chromosomes are not likely to be detected at the stage of DSB formation, because this requires strand invasion. However, the heterozygosity has a tremendous impact on crossover distribution by influencing crossover/non-crossover decision. Detection of mismatches during meiotic recombination is possible thanks to mismatch-repair system (MMR) (Manhart and Alani, 2016). In this pathway, heterodimers of MutS homologs (MSH2-MSH3, MSH2-MSH6 or MSH2-MSH7) bind DNA, detect mismatches and recruit heterodimers of MutL homologs (MLH1-MLH3 and MLH1-PMS1) in an ATP-dependent reaction. MLH1-PMS1 complex exhibits strong anti-crossover function, while MLH1-MLH3 complex, in combination with SGS1 and EXO1, is able to resolve double Holliday junctions as crossovers (Börner et al., 2004; Bzymek et al., 2010; Manhart and Alani, 2016). This happens via the major crossover pathway ZMM, which in Arabidopsis involves MSH4, MSH5, MER3, HEI10, ZIP4, SHOC1, PTD) (Copenhaver et al., 2002; Higgins et al., 2004, 2008b; Chen et al., 2005; Macaisne et al., 2008; Chelysheva et al., 2012, 2007). In plants, similarly to many other eukaryotes, strand invasions, which are not resolved by ZMM pathway, can be also repaired by the minor crossover pathway (Berchowitz et al., 2007; Higgins et al., 2008a). This relies on the partially redundant structure-specific nucleases and is not biased toward crossover (De Los Santos et al., 2003; Mercier et al., 2015; Wang and Copenhaver, 2018). The exact mechanism for the ZMM crossover bias is currently not known.

### Chromosomal Scale

Early works in bacteria indicated that the recombinant frequencies between mismatched substrates were much lower than those of perfectly matched substrates (Claverys and Lacks, 1986; Shen and Huang, 1986), and that the MMR system establishes a genetic barrier during recombination of diverged sequences (Rayssiguier et al., 1989; Shen and Huang, 1989; Matic et al., 1995). Several studies have demonstrated that also in budding yeast decreased sequence homology between chromosomes significantly reduces meiotic recombination (Nilsson-Tillgren et al., 1981, 1986; Hunter et al., 1996). In the study, where the cross between two *Saccharomyces* species were investigated, Hunter et al. (1996) found that the resulting interspecific hybrid gave high rate of aneuploidy and low levels of meiotic recombination, but when the same experiment was repeated in the genetic background of MutH and MutL homolog mutants, *msh2* and *pms1*, an increase in recombination and reduction in aneuploidy was observed. Furthermore, in a *S. cerevisiae* diploid with one copy of chromosome III from *Saccharomyces paradoxus*, the mismatch repair (MMR)-dependent inhibition of recombination between the homeologous (i.e., heterozygous) chromosomes was also observed, so that in *pms1* and *msh2* mutants, the recombination was increased between the two chromosomes III leading to reduction in non-disjunction of this chromosome (Chambers et al., 1996). As only one of the 12 yeast chromosomes was homeologous, it is unlikely that the effect observed could be due to any potential *trans*-acting modifiers. Those studies confirmed that in yeast, similarly to bacteria, heterozygosity suppresses crossover, and that the MMR system acts as a genetic barrier for meiotic recombination between not-perfectly matching chromosomes.

In plants, analysis of the effect of heterozygosity on meiotic recombination in the chromosomal scale is limited due to existence of *trans*-acting modifiers, which could affect recombination in hybrids. This could lead to results that are difficult to interpret. For instance, in *A. thaliana* several studies of meiotic recombination in F_1_ and F_2_ plants show extensive variation in crossover numbers that does not correlate with sequence differences between parental accessions (Alonso-Blanco et al., 1998; Simon et al., 2008; Salomé et al., 2011; Ziolkowski et al., 2015). Similarly, no such correlation has been reported in maize (Beavis and Grant, 1991; Bauer et al., 2013). This problem could be however partially overcome when chromosome substitution lines would be used for comparison. In chromosome substitution lines a pair of chromosomes in one line or species is replaced by a homeologous pair from another variety/species. Sets of chromosome substitution lines were developed especially for plant crops, though direct comparison of crossover frequencies was not frequent. In tomato, interspecific hybrid between *Lycopersicon esculentum* and *Solanum lycopersicoides* shows ca. 27% reduction in meiotic recombination (Chetelat et al., 2000). Interestingly, heterozygous substitution lines containing a single *S. lycopersicoides* chromosome bred into *S. lycopersicum* recombine at less than 50% of the rate observed for the same chromosome in the F_1_ hybrid (Ji and Chetelat, 2003). The fact that most of *L. esculentum* and *S. lycopersicoides* chromosomes can be distinguished using genomic in situ hybridization (GISH) suggests that the two genomes have diverged substantially in terms of dispersed repetitive sequences. One of those substitution lines was also analyzed in a background, where MMR system was not fully functional, and this resulted in an increase of crossover frequency (average 17.8% increase when compared to wild type) (Tam et al., 2011). Similar results were obtained in *Arabidopsis thaliana*, where Col x Ler inter-accession hybrid in *msh2* mutant exhibited 1.4-fold increase of CO rate when compared to wild type (Emmanuel et al., 2006). This indicates that observed suppression of recombination is mostly due to heterozygous state in *cis*.

Suppression of recombination in polymorphic regions is believed to be important for prevention of deleterious ectopic recombination between repetitive sequences in a genome (Modrich and Lahue, 1996; Borts et al., 2000; Evans and Alani, 2000). In general, non-allelic copies of repetitive sequences rapidly accumulate mutations, which help to distinguish them from allelic copies. The conservancy of MMR system and similar effects of its malfunction in different organisms suggest that this effect is universal across eukaryotes.

### Local Effects, Hotspot Level

The effect of heterozygosity on meiotic crossover frequency was also analyzed at the recombination-hotspot scale. Borts and Haber (1987) tested the effect of heterozygosity on meiotic recombination products in an artificial *MAT-pBR322-URA3-MAT* interval. By using yeast strains that contain mismatches within this hotspot (about 0.1% divergence between strains) they showed that the number of crossover events was reduced from 23.4 to 10.1% when compared to fully homozygous strains, and there was a corresponding increase in aberrant events, as detected with the flanking markers. In *pms1* mutant recombination was restored, leading the authors to propose that independent repair of these widely spaced mismatches might result in the formation of new double-strand breaks that could in turn stimulate a second round of recombination (Borts et al., 1990). These events were detected because of the presence of flanking repeated *MAT* sequences and this is a likely reason why they were not detected in other experimental systems (Symington and Petes, 1988; Malone et al., 1994). In mice, highly polymorphic *A3* hotspot was repaired mostly via interhomolog NCO pathway and CO refractory zone corresponded to a region containing three indels (Cole et al., 2010). This indicates that also in mammals, polymorphism does not influence DSB formation, however, it affects selection of a repair pathway.

In plants, major work on this subject has been made in maize, where the characterization of strong recombination hotspots *a1-sh2* and *bronze* (Dooner, 1986; Yao et al., 2002), and high genetic diversity between different maize inbred lines (Fu and Dooner, 2002; Lai et al., 2010) provided a perfect experimental system. The 130-kb of the *a1-sh2* region exhibits meiotic recombination rate between 0 and 11 cM/Mb, which is significantly more than region average (0.0087 cM/Mb) (Yao et al., 2002). The 1.5-kb long *bronze* locus has a recombination frequency at least 100 times higher than the average for the maize genome (Dooner, 1986; Dooner and Martínez-Férez, 1997). Both intervals are highly polymorphic between maize haplotypes and reveal both genic and non-genic collinearities (Fu and Dooner, 2002; Yao et al., 2002; Brunner et al., 2005). Even bigger differences have been observed when compared to teosinte haplotypes, which is considered as a wild progenitor of maize (Yao and Schnable, 2005).

In most of the studies the authors concluded that recombination is suppressed by polymorphisms (Dooner and Martínez-Férez, 1997; Yao and Schnable, 2005). Dooner and Martínez-Férez (1997) in an experimental setup, where crosses between maize lines with different number of polymorphisms with a tester line were used, observed a good negative correlation with recombination rate. Similar effects were observed in experiments, where neighboring regions where compared: subintervals that exhibit higher recombination rates per megabase than their juxtaposed subintervals, also exhibit lower levels of polymorphisms (Yao and Schnable, 2005). Less clear relationship was observed for non-adjacent intervals. The general problem with interpretation of those data is that different types of polymorphisms may affect recombination to a different extent, especially SNPs and indels cannot be treated in the same way. In case of experiments with *a1-sh2* and *bronze* hotspots, large transposable elements existing in those lines significantly reduce recombination (Fu et al., 2002; Dooner and He, 2008; He and Dooner, 2009). For instance, a 26 kb retrotransposon cluster located nearby *bronze* locus suppresses crossover in this hotspot by a factor of two (Dooner and He, 2008), and haplotype structure as defined by the presence of helitrons and retrotransposons in this locus, strongly inhibited occurrence of recombination in heterozygous plants (He and Dooner, 2009).

**FIGURE 3:**
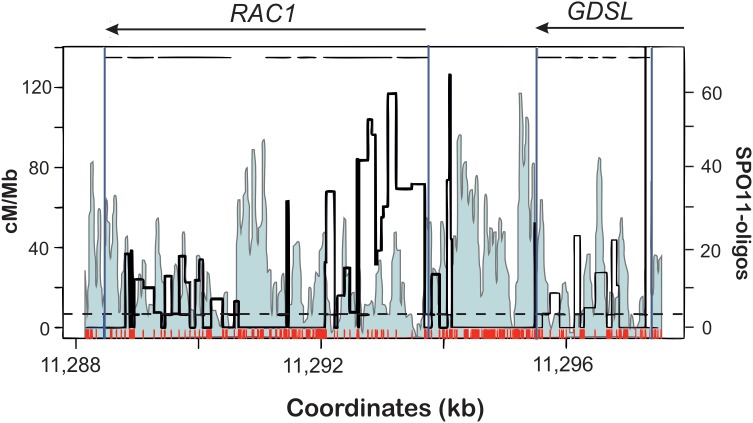
Distributions of DSBs (SPO11-1-oligos; light blue profile) and COs (cM/Mb; thick black line) within the *RAC1-GDSL* hotspot in *A. thaliana*. Polymorphism between Col and Ler accessions used for CO mapping is depicted as red ticks. Gene orientation and exon-intron structure is shown at the top of the plot. Note overrepresentation of DSBs at 5′-ends, 3′-ends of the genes, and within some introns. Crossovers appear mostly in SNP-free fragments of the hotspot. Modified from Choi et al. (2018).

In *A. thaliana*, conducting the crossover frequency studies at the hotspot level were not possible for a long time due to the lack of morphological markers similar to those used in maize. However, recent development of pollen typing technique enabled to overcome this limitation (Drouaud and Mézard, 2011; Yelina et al., 2012; Choi et al., 2013; Drouaud et al., 2013). Pollen typing, similarly to sperm typing developed for mammals is based on an allele-specific amplification of a hotspot region from post-meiotic gametes. Subsequent sequencing of the PCR products enables precise determination of recombination breakpoints in regards to polymorphic regions, even though in pollen typing comparison of crossover landscape with completely homozygous line is not possible. Analysis of crossover distribution in relation to SNPs shows that polymorphism suppresses crossover formation at the hotspot scale in a way similar to other eukaryotes (Choi et al., 2013, 2016; Yelina et al., 2015, 2012; Ziolkowski and Henderson, 2017; Lawrence et al., 2018). NCO analysis, as being technically more challenging, was performed only in one study (Drouaud et al., 2013). In other cases, the authors studied only crossover events, therefore it is difficult to conclude on DSB distribution at the hotspots. Fortunately, for direct comparison we can use data from SPO11-oligo sequencing to observe the pattern of DSBs in the crossover hotspots (Choi et al., 2018). Distribution of CO events clearly shows inhibition at polymorphic sites (Choi et al., 2013, 2016). This is especially visible for the highly polymorphic hotspot *RAC1-GDSL* (Figure 3). As expected, distribution of DSBs is not affected by SNPs and actually the levels of SPO11-oligos are elevated in SNP-rich regions. This suggests that the polymorphism resulted from recombination-associated mutations.

**FIGURE 4:**
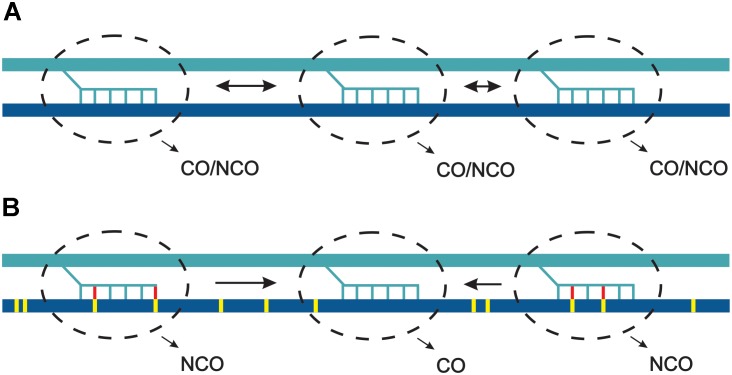
Model of competition between hotspots in response to sequence heterology. **(A)** Three hotspots (dashed line ovals) become activated and involved in strand invasion into the two homologous chromosomes, which are identical regarding the sequence. Due to scarcity of crossover, they all compete and have similar chances for developing into a crossover. **(B)** The two homologous chromosomes differ at single base pairs (yellow ticks). During strand invasion mismatches are detected by MMR (yellow-red ticks) and develop into non-crossovers. In consequence, the perfectly matched invasions have higher chance for becoming crossovers. For simplicity, one direction of strand invasion was shown.

Assuming polymorphism-independent distribution of DSB within hotspots, we can conclude that at the kilobase scale polymorphic sites cause inhibition of CO pathways and are repaired mostly by NCOs. If DSB sites compete for CO factors, polymorphism inhibiting CO would lead to recombinational hyperactivity of some hotspots. In other words, polymorphism would act inhibitory at the single hotspot scale, but increase a chance of adjacent polymorphism-free hotspot for entering a crossover repair pathway (Figure 4).

### Sensitivity of Different Recombination Pathways Toward Heterozygosity

As it was already mentioned, at least two meiotic crossover pathways exist in most eukaryotes including plants (Higgins et al., 2004; Mercier et al., 2005). The major pathway in *A. thaliana* is responsible for about 85% of crossover events (Chelysheva et al., 2007), is interference-sensitive, and depends on a group of proteins that are collectively called ZMM (Copenhaver et al., 2002; Higgins et al., 2004, 2008b; Chen et al., 2005; Macaisne et al., 2008; Chelysheva et al., 2012, 2007). The remaining crossovers are non-interfering, randomly distributed along the chromosomes, and are dependent on recombinases such as MUS81 that are not meiosis-specific and that have important roles also in somatic cells (Berchowitz et al., 2007; Higgins et al., 2008a). In wild type, the recombination intermediates in non-interfering DSB repair are mostly directed toward NCO pathway by the FANCM helicase (Crismani et al., 2012; Knoll et al., 2012) and the BLM/SGS1 helicase homologs RECQ4A/B (Séguéla-Arnaud et al., 2015, 2017). In addition, strand invasion step is affected by the AAA-ATPase FIGL1, which hinders the interaction with a homologous chromosome (Girard et al., 2015; Fernandes et al., 2018a). Dramatic increases in crossover frequency are observed in mutants of those genes.

The class I and II pathways have been compared with the respect to sensitivity to polymorphism in the chromosomal region scale in *Arabidopsis thaliana*. Due to lack of proper non-interfering mutants (Mercier et al., 2015) class II behavior can be analyzed only indirectly, e.g., by using *fancm* mutant, where non-interfering repair is directed toward crossover (Crismani et al., 2012; Knoll et al., 2012). Ziolkowski et al. (2015) reported that in *fancm* background very little increase is observed in recombination frequency within chromosomal regions in heterozygous state when compared to wild type Arabidopsis. In *fancm zip4* double mutant a significant reduction in crossover rate was observed for heterozygous regions, even though the same mutant in homozygous regions shows a dramatic increase. Consistently with this, an increased interference in heterozygous regions is observed in wild type plants (Ziolkowski et al., 2015). Although no direct analysis on how the level of polymorphisms affects the inhibition was carried out, highly polymorphic pericentromeric regions exhibited higher suppressive effect on class II crossover frequency than less polymorphic subtelomeric regions. The authors concluded that both crossover pathways show opposite sensitivity toward heterozygosity, with non-interfering pathways being unable to successfully repair DSBs in such regions, at least in *fancm* background. Girard et al. (2015) also observed no increase in crossover rate in *fancm* mutant in hybrids, but a significant increase was observed in *fancm figl1* double mutant when compared to either wild type or *figl1*. FIGL1 is a protein suggested to limit strand invasion step during recombination by regulation of DMC1 and RAD51 proteins (Girard et al., 2015; Fernandes et al., 2018a). Thus, the authors concluded that in the absence of *FIGL1* protein the non-interfering *FANCM*-dependent pathway may successfully repair heterozygous chromosomal regions by crossover. This suggests existence of another unknown mechanism, which impairs the anti-crossover FANCM activity in hybrids (Girard et al., 2015).

In a more recent study Fernandes et al. (2018b) investigated the accumulated effect of *A. thaliana* mutants in all three anti-recombinational pathways, i.e., *recq4, figl1* and *fancm*, and observed extensive increases in CO rates in inter-accession Col x Ler crosses. However, only marginal increase in CO rate was observed for pericentromeric regions. The authors proposed that this may be due to limited accessibility of pericentromeric chromatin for SPO11, which results in lack of recombination initiation sites (Fernandes et al., 2018b). This explanation seems probable when we consider recent finding of drop in DSBs in Arabidopsis pericentromeres (Choi et al., 2018). Moreover, a strong anticorrelation between recombination and SNP density was reported in *recq4 figl1*, which was not observed in wild type. This implicates inhibiting effect of polymorphism on crossover rate (Fernandes et al., 2018b). Supporting this observation, significantly lower CO levels where observed in the middle of chromosome 1 right arm, which corresponds to significant elevation of polymorphisms (Ziolkowski et al., 2009; Choi et al., 2016). Therefore, lack of extra COs in pericentromeric regions may be partially due to elevated polymorphisms which seems to discourage CO repair pathway (Fernandes et al., 2018b). To verify this hypothesis an experiment including heterozygosity-homozygosity juxtaposition would be necessary. Further experiments involving the use of proper class II crossover mutants would be required to fully understand the polymorphism-sensitivity of both crossover pathways.

### Juxtaposition of Heterozygous and Homozygous Regions Changes the Chromosomal Redistribution of Crossover

**FIGURE 5:**
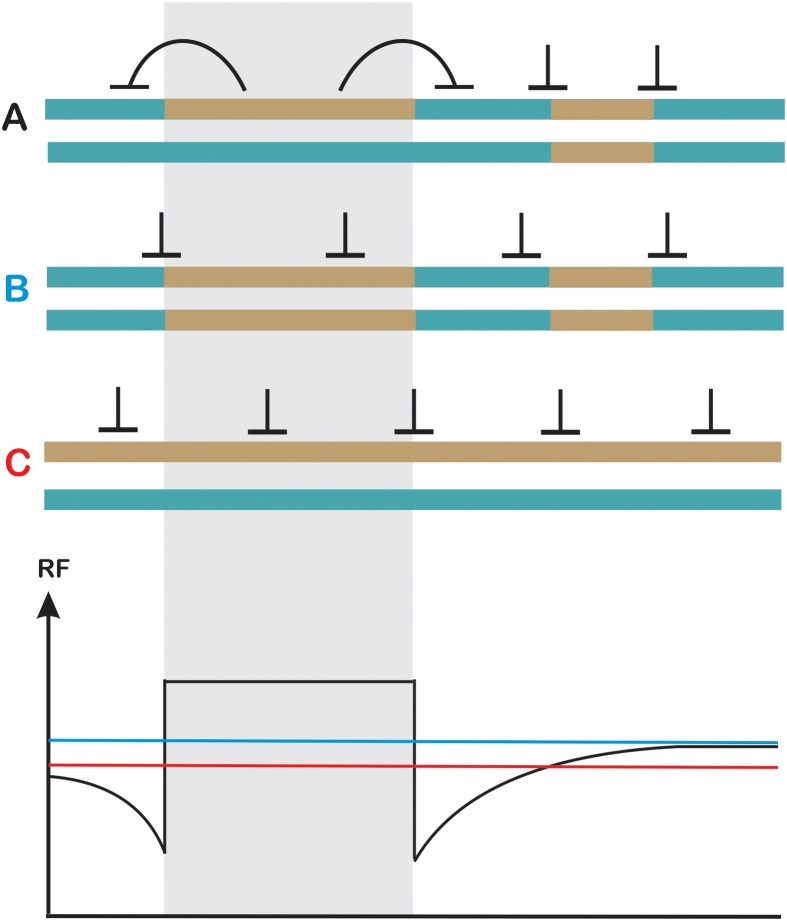
Heterozygosity juxtaposition effect. Two homologous chromosomes in **(A–C)** differ in the pattern of heterozygosity (turquoise and dark-yellow). **(A)** Crossover levels get elevated in a heterozygous region at the expense of adjacent homozygous regions on the same chromosome (in *cis*). **(B)** Crossovers are evenly spaced in fully homozygous chromosomes **(C)** Crossovers are evenly spaced in fully heterozygous chromosomes although a reduction in recombination frequency at the chromosome scale is observed. Other effects, which could affect crossover spatial distribution were not shown for simplicity. Recombination levels are schematically shown on the lower panel using the color code for **(A–C)**.

The widely documented suppression of crossover frequency at the hotspot level contradicts with the data collected at the chromosomal scale in *A. thaliana*, when homozygous and heterozygous segments were juxtaposed (Ziolkowski et al., 2015). In such experimental setup a reciprocal crossover increases in heterozygous and decreases in homozygous regions were observed (Figure 5). The total number of crossovers measured by chiasmata counting were not changed, consistent with homeostatic regulation. This phenomenon seems to be independent of chromosomal location as it was shown for two different chromosomes and for both subtelomeric and pericentromeric intervals, and was observed in different *A. thaliana* crosses (I. R. Henderson, personal communication) Analysis in *fancm, zip4* and *fancm zip4* mutant background provided strong evidence that the process is interference-dependent.

The mechanisms by which juxtaposition effect is executed is not understood, however, it must involve detection of mismatches by MMR proteins, as the effect is dependent mostly on ZMM pathway. It is possible that specific signaling between MMR components and ATM/ATR pathway results in additional DSBs being formed in the region, and this can also include some type of DSB site competition. It is also currently unknown whether this phenomenon is unique to *Arabidopsis*, or is a general feature of interference-dependent crossover pathway in eukaryotes. Conservation of major components of meiotic DSBs formation and interference-dependent repair pathways suggests that it may exist in other organisms, especially in self-pollinating plant species where situations of adjacent homozygous and heterozygous regions are common. The biological meaning of this process would be to increase the chance to generate novel combinations of genetic material: COs occurring in homozygous regions result in reestablishing parental haplotypes in the next generation, while stimulating recombination in heterozygous segments always result in some new allele assemblies.

## Concluding Remarks and Perspectives

Recent discoveries in the field of meiotic recombination significantly changed our understanding of processes responsible for shaping the genome. However, substantial differences have been spotted between mammals and plants. In mammals, PRDM9 histone methyltransferase plays a key role in defining crossover sites, whilst plants the distribution of recombination is dependent on a large number of subtle features, both at the level of genetics and chromatin structure. For instance, it is currently unknown whether H3K4me3 plays a similar function in recombination hotspot tethering to the chromatin loops in plants, as it was shown for budding yeast and animals, as the data are inconsistent. From this perspective further work is needed to define the relationships between particular levels and find rules responsible for priority of some factors over the others.

In comparison with recombination initiation sites, additional regulatory levels of crossover distribution result from CO/NCO decision. Recent developments in plants, especially approaches to asses DSB levels and fine-scale crossover mapping (He et al., 2017; Choi et al., 2018), show that those factors may have also epigenetic origin. The major problem, which researchers meet in their trials to decipher epigenetic factors, lies in the extensive crosstalk between different epigenetic modifications and the fact that they operate on a global scale. Therefore, new targeted approaches will be required to investigate effects of particular alterations locally and at the hotspot scale. Directing particular modifications to specific chromosomal locations, together with targeting recombination events, possibly using CRISPR-dCas9 technology, may provide an attractive strategy for this purpose and should lead to further fascinating discoveries.

Another interesting topic, which requires further investigation, refers to interactions between homologs chromosomes, where local differences in DNA sequence, and probably also local chromatin states, affect the outcomes of strand invasion. This is particularly interesting in self-pollinating plants, which are characterized by a high level of sequence homozygosity. Their rare outcrossing has a result in the existence of heterozygous regions juxtaposed to homozygous ones on the same chromosome, and thereby creates novel chances for genome evolution (Ziolkowski et al., 2015). It would be interesting to investigate the mechanism responsible for these *cis* effects on crossover stimulation. In this context, questions about potential effects of “epigenetic heterozygosity” and competition between recombination hotspots would be intriguing to answer. Those findings, along with a recent progress in the identification of *trans*-acting factors responsible for crossover distribution (Ziolkowski et al., 2017; Fernandes et al., 2018b) opens new perspectives for developing novel breeding strategies (Choi, 2017; Lambing and Heckmann, 2018).

## Author Contributions

All authors listed have made a substantial, direct and intellectual contribution to the work, and approved it for publication.

## Conflict of Interest Statement

The authors declare that the research was conducted in the absence of any commercial or financial relationships that could be construed as a potential conflict of interest.
